# Global Comparison of Stability Testing Parameters and Testing Methods for Finished Herbal Products

**DOI:** 10.1155/2019/7348929

**Published:** 2019-10-20

**Authors:** Jung-Hoon Kim, Kyungjin Lee, Ui Min Jerng, Goya Choi

**Affiliations:** ^1^Division of Pharmacology, School of Korean Medicine, Pusan National University, Yangsan 50612, Republic of Korea; ^2^Department of Herbal Pharmacology, College of Korean Medicine, Kyung Hee University, Seoul 02447, Republic of Korea; ^3^Department of Internal Medicine, College of Korean Medicine, Sangji University, Wonju 26339, Republic of Korea; ^4^Herbal Medicine Resources Research Center, Korea Institute of Oriental Medicine, Daejeon 34054, Republic of Korea

## Abstract

Quality consistencies of drug products are essential to guarantee expected therapeutic activities, and achieving consistent qualities for herbal products is challenging because of their physicochemical complexities and inherent variabilities. Regulatory authorities worldwide have issued regulations or guidelines for stability testing parameters and testing procedures for herbal products stored in proposed conditions. These testing parameters and methods for finished herbal products are detailed in the guidelines and regulations issued by 5 global authorities and 15 countries, that is, the Association of Southeast Asian Nations (ASEAN), the Eurasian Economic Commission (EEC), the European Medicines Agency (EMA), the International Council for Harmonisation of Technical Requirements for Pharmaceuticals for Human Use (ICH), the World Health Organization (WHO), Australia, Brazil, Canada, China, Egypt, Hong Kong, India, Japan, Kenya, Republic of Korea, the Philippines, Qatar, Switzerland, USA, and Zambia. Physical, chemical, and biological stability tests were compared between different dosage forms, and the testing conditions (temperature and relative humidity) used for long-term, accelerated, or intermediate testing were included in the guidelines and regulations. Comparisons of global regulations and guidelines addressing stability testing are fundamental for the international harmonization of herbal product quality assessments. This review aids understanding of the global situation regarding the testing of herbal product quality with respect to storages.

## 1. Introduction

The maintenance of herbal product quality during storage is critical for guaranteeing therapeutic activity. Stability testing is used to evaluate how herbal products retain their properties under specified storage conditions stressed by heat, moisture, light, oxygen, various physical and chemical conditions (e.g., vibration or freezing), and container-related factors [1, 2]. Herbal products are produced in various dosage forms (e.g., tablets, powders, or liquids for oral administration or as creams for external application), and thus, stability testing of various dosage forms requires appropriate methods.

The stabilities of finished herbal products can be determined by testing for properties susceptible to storage conditions and include physical (organoleptic characteristics, physical condition, particle size, etc.), chemical (assays of active components, pH, identification, etc.), microbial, and toxicological properties. These properties can all affect the qualities, safeties, or the efficacies of herbal products, and thus, the shelf lives of herbal products should be determined by stability testing [3].

Furthermore, different stability protocols are used in different countries as herbal products are generally developed to meet national regulations. Global harmonization of stability testing has been recently emphasized in the context of herbal drug development, but the adoption of international standards can only be achieved by sharing national experiences and information [4].

Therefore, in the present study, we detail the stability testing parameters and methods used for herbal products of different dosage forms as detailed by the guidelines and regulations issued by global authorities including the Association of Southeast Asian Nations (ASEAN), the Eurasian Economic Commission (EEC), the European Medicines Agency (EMA), the International Council for Harmonisation of Technical Requirements for Pharmaceuticals for Human Use (ICH), and the World Health Organization (WHO), and those issued by individual countries such as Australia, Brazil, Canada, China (and Hong Kong), Egypt, India, Japan, Kenya, Republic of Korea, the Philippines, Qatar, Switzerland, USA, and Zambia.

## 2. An Overview of Stability Testing of Herbal Dosage Forms

We searched guidelines and regulations from global authorities and countries where the stability testing is required for quality maintenance of herbal products (or final herbal preparations). Testing parameters (or quality testing indicators) were crucially provided according to different dosage forms of herbal products. Conditions for long-term, accelerated, and intermediate testing (e.g., storage temperature, relative humidity, period, etc.) were compared among global authorities and countries. Guidelines and regulations which requires quality of only chemical drugs were excluded.

### 2.1. Stability Testing Parameters

#### 2.1.1. ASEAN

ASEAN issues the stability testing guidelines to ensure quality maintenance of *final herbal products* (*traditional medicines*) in specified packages that apply to recommended storage conditions and times. These guidelines require that physical, chemical, and microbiological parameters of finished products should be addressed. The parameters for dosage forms are as follows: oral powders (organoleptic characteristics, assay, water content, and microbial content); hard capsules (organoleptic characteristics, assay, dissolution, disintegration, water content, and microbial content); soft capsules (organoleptic characteristics, assay, dissolution, disintegration, and microbial content); tablets (coated and uncoated; organoleptic characteristics, assay, hardness, friability, dissolution, disintegration, water content, and microbial content); pills and pellets (coated and uncoated; organoleptic characteristics, assay, dissolution, disintegration, water content, and microbial content); suspensions (organoleptic characteristics, assay, viscosity, pH, microbial content, granules, or particle-size variation, and resuspendability); solutions (organoleptic characteristics, assay, viscosity, pH, and microbial content); emulsions (organoleptic characteristics, assay, viscosity, pH, and microbial content); semisolid preparations (ointment, cream, gel, lotion, and paste; organoleptic characteristics, assay, viscosity, pH, and microbial content); plasters (organoleptic characteristics, assay, microbial content, and adhesiveness); granules (organoleptic characteristics, assay, water content, microbial content, and granules or particle-size variation); herbal infusion bags and herbal tea bags (organoleptic characteristics, assay, water content, and microbial content); and pastilles (organoleptic characteristics, assay, water content, and microbial content) [5].

#### 2.1.2. The EEC

The EEC requires storage stability studies to be conducted in *herbal medicinal preparations* for registration in accordance with regulations for pharmaceutical substances and medicinal products [6]. Stability testing involves determining values of physical, chemical, biological, microbiological indicators, preservative contents (e.g., antioxidants and antimicrobial preservatives), and delivery device functions (e.g., dose delivery system) [7]. The regulations require that all medicinal products should be evaluated in terms of appearance, active ingredient, degradation products, preservative, and antioxidant contents.

In addition, the EEC regulations require dosage form be tested as follows: pills (dissolution^*∗*^, disintegration^*∗*^, water content, and resistance to abrasion for pills without a shell); hard gelatin capsules (fragility, dissolution^*∗*^, disintegration^*∗*^, water content, and microbiological purity); soft gelatin capsules (dissolution^*∗*^, disintegration^*∗*^, microbiological purity, pH, hermeticity, and adhesion); oral emulsions, suspensions, and solutions (sludge formation, pH, viscosity, extractable substances, and microbiological purity) with additional testing parameters required for solutions (transparency), suspensions (dispersion, rheological properties, average particle-size/distribution, polymorphic transformations^*∗*^, and the interconversion of polymorphs), and emulsions (phase separation and average size and distribution of the dispersed globules); powders and granules for oral solutions or suspensions (water content and recovery); metered dose inhalers (uniformity of dose content, number of activations of the container valve, aerodynamic particle-size distribution, microscopic evaluation, water content, hermeticity, microbial contamination, valve delivery or injection weight, weight loss, pump delivery, foreign mechanical inclusions, and substances extracted and discharged from plastic and elastomeric components of the container, closure, and pump); suspension aerosols (microscopic analysis of the appearance of valve components and container contents, large inclusions, changes in the morphology of particles of the pharmaceutical substance, the agglomerates, crystals, foreign mechanical inclusions, and corrosion of the inner surface of the container and the wear of the spacer); nasal sprays (transparency for solutions, microbiological purity, pH value, mechanical inclusions, uniformity of active ingredient content in one injection, size distribution of drops or particles, weight loss, pump delivery, microscopic evaluation of suspensions, foreign mechanical inclusions, and substances extracted and discharged from plastic and elastomeric components of the container, closure, and pump); dosage forms for topical (external) use, eye and ear applications including ointments, creams, pastes, gels, solutions, eye drops, and sprays for external use (transparency, homogeneity, pH value, ability to resuspend for lotions, thickness, viscosity, particle-size distribution for suspensions, microbiological purity, and weight loss^*∗*^) including additional parameters for eye and ear medicinal products including creams, ointments, solutions, and suspensions (sterility, mechanical inclusions, and extractable volume) and sprays for external use (pressure, weight loss, total extractable weight, speed of delivery, microbiological purity, spraying performance, water content, and particle-size distribution for suspensions); suppositories (degree of softening, and disintegration and dissolution at a temperature of 37°C); parenteral medicinal products (color, transparency for solutions, mechanical inclusions, pH value, sterility, pyrogenicity, endotoxin content, and volume); and transdermal patches (release rate *in vitro*, hermeticity, microbiological purity/sterility, ungluing strength, and shear adhesion) [7]. Asterisks indicate optional test parameters.

#### 2.1.3. The EMA

The EMA requires product-specific storage stability testing of herbal medicinal products for quality assurance, as detailed in the Note for guidance on stability testing of new drug substances and products (CPMP/ICH/2736/99), the “Guideline on stability testing of new veterinary drug substances and medicinal products (CVMP/VICH/899/99),” and the “Guideline on stability testing of existing active substances and related finished products (CPMP/QWP/122/02 and EMEA/CVMP/846/99)” [8].

According to the EMA guidelines, all herbal medicinal products should be tested for the compliance with specifications including descriptions, identifications, assay, impurities, and microbial limits. The following testing parameters are also specified: tablets (coated and uncoated) and hard capsules (dissolution, disintegration, hardness, friability, uniformity of mass, water content, and microbial limits); oral suspension (uniformity of mass, pH, microbial limits, antimicrobial preservative content, antioxidant preservative content, extractables, alcohol content, dissolution (for oral suspensions), and resuspension (for dry powder products), particle-size distribution, redispersibility, rheological properties for relatively viscous solutions or suspensions, viscosity, specific gravity (for oral suspensions, relatively viscous, or nonaqueous solutions), reconstitution time, and water content); and herbal teas (loss on drying, identification, purity, uniformity of mass or average mass of the sachet, assay, particle size, and microbial quality or microbial limit testing) [8, 9].

#### 2.1.4. ICH

ICH guidelines provide general requirements for the storage stability testing of new drug products that cover chemical substances with respect to description, identification, assay, and impurity contents [10]. However, we consider ICH guidelines are also applicable to herbal products as global documents regulated by EMA, Australia, Japan, or Switzerland are conducted in accordance with ICH guidelines. The parameters for stability testing of specific dosage forms are as follows: tablets (coated and uncoated) and hard capsules (dissolution, disintegration, hardness, friability, uniformity of dosage units, water content, and microbial limits); oral liquids (uniformity of dosage units, pH, microbial limits, antimicrobial and antioxidant preservative content, extractables, alcohol content, dissolution, particle-size distribution in oral suspensions, redispersibility for oral suspensions, rheological properties for relatively viscous solutions or suspensions, reconstitution time, and water content); parenteral drug products (uniformity of dosage units, pH, sterility, endotoxins, pyrogens, particulate matter, water content, antimicrobial and antioxidant preservative content, extractables, functionality testing of delivery systems including prefilled syringes, autoinjector cartridges, or the equivalent, osmolarity, particle-size distribution for injectable suspensions, redispersibility, and reconstitution time) [10].

#### 2.1.5. WHO

The WHO expert committee publishes technical reports annually on specifications for pharmaceutical preparations and guidelines on good herbal processing practices for herbal medicines (Annex 1) and on stability testing for active pharmaceutical ingredients and finished pharmaceutical products (Annex 10) [3].

General requirements of the stabilities of *finished pharmaceutical products* include appearance, assay, and degradation products and preservative and antioxidant content. Specific parameters are also provided according to dosage forms of the product, that is, as liquids, solids, or others [3].

Liquid herbal dosage forms include fluid extracts, decoctions, infusions, tinctures, syrups, and oral solutions, which are tested for precipitate formation, clarity, pH, viscosity, extractables, and microbial contamination level. Oral suspensions are tested for precipitate formation, clarity, pH, viscosity, extractables, microbial contamination level, dispersibility, rheological properties, mean size or distribution of particles, and polymorphic conversion. Oral emulsions are tested for precipitate formation, clarity, pH, viscosity, extractables, microbial contamination level, phase separation, and globule mean size or distribution. For aromatic water, and powders or granules for oral solutions or suspensions, water content and reconstitution time are tested [3].

Solid herbal dosage forms include herbal tea bags, plant powders, dry extract powders, granules, pills, hard gelatin capsules, soft gelatin capsules, tablets, and lozenges. Hard gelatin capsules are tested for brittleness, dissolution, disintegration, water content, and microbial contamination level. Soft gelatin capsules are tested for dissolution, disintegration, microbial contamination level, pH, leakage, and pellicle formation. Tablets are tested for dissolution, disintegration, water content, hardness, and friability [3].

Other dosage forms include ointments, creams, and salves which are tested for clarity, homogeneity, pH, suspendability (for lotions), consistency, viscosity, particle-size distribution (for suspensions), microbial contamination level, sterility, and weight loss. Ophthalmic and otic products (e.g., creams, ointments, solutions, and suspensions) are tested for sterility, particulate matter, and extractable volume. Inhalers are tested for dose content uniformity, labelled number of medication actuations per container that meet stated dose delivery, aerodynamic particle-size distribution, microscopic evaluation, water content, leak rate, level of microbial contamination, valve delivery or shot weight, extractables or leachables from plastic and elastomeric components, weight loss, pump delivery, foreign particulate matter, extractables or leachables from plastic, and elastomeric components of the container, closure, and pump. Plasters and patches are tested for *in vitro* release rates, leakage, level of microbial contamination, sterility, peel strength, and adhesive forces. Medicated oils are also included in other dosage forms, but testing parameters are not provided [3].

#### 2.1.6. Australia

The Australian government provides mandatory guidelines for the stability testing of *complementary medicines* in different dosage forms as follows: solutions, suspensions, creams, ointments, tablets (produced by direct compression), tablets (produced by granulation), capsules (two-piece, produced by dry mixing), capsules (two pieces, produced by granulation), soft capsules (soft gels) containing solutions, soft capsules (soft gels) containing suspensions, and powder mixes [11]. Stability testing for determining shelf lives and recommended storage conditions is performed as described in the EMA guideline, “Guideline on stability testing: stability testing of existing active substances and related finished products (CPMP/QWP/122/02 rev 1 corr)” [12].

#### 2.1.7. Brazil

The Brazilian Health Regulatory Agency (ANVISA) adopts the position that stabilities herbal products depending on environmental factors (temperature, humidity, and light) as well as product-related parameters (physical and chemical properties of active substances and excipients, pharmaceutical form, product composition, manufacture, and properties of packaging materials). Stability studies should be of an accelerated nature and were conducted long term to establish shelf-life and suitable storage conditions [13].

ANVISA provides stability testing parameters of *pharmaceutical form for phytotherapy* as follows: pills and tablets (description, disintegration, dissolution, hardness, water content, friability, uniformity of dose unit, average weight, and active component content); capsules (description, disintegration, dissolution, water content, uniformity of dose unit, average weight, and active component content); granules (description, particle size, water content, friability, fluidity, bulk density, uniformity of dose unit, average weight, and active component content); tinctures and syrups (description, pH, viscosity, relative density, sucrose content, uniformity of dose unit, and active component content); semisolids (description, pH, uniformity of dose unit, average weight, phase separation, and active component content); transdermal adhesives (description, uniformity of dose unit, adhesive strength, tensile strength, and active component content); intravaginal suppositories (description, disintegration, dissolution, pH, softening temperature, uniformity of dose unit, average weight, and active component content); and medicated soaps (description, pH, uniformity of dose unit, average weight, and active component content). In addition, microbial testing is required for all dosage forms [14].

#### 2.1.8. Canada

Canadian government requires storage stability testing for *natural and nonprescription health products* to determine shelf lives after packing and storage conditions. These tests address purity, physical characteristics, level of medicinal ingredients quantity per dosage unit, and potency [15].

Canadian government prescribes guidelines that provide physical testing parameters for different dosage forms, as follows: tablets, caplets, and capsules for immediate release (description, disintegration, and weight variation or average weight); rapidly dissolving tablets (description, dissolution, and weight variation or average weight); tablets and capsules for extended release, combined release, or timed release (description, dissolution, weight variation or average weight, and uniformity of dosage unit); tablets and capsules for delayed release, including enteric coated tablets and capsules (description, disintegration, and weight variation or average weight); oral solutions and suspensions (description and preservative efficacy); topical preparations (description and preservative efficacy); transdermal patches (description, uniformity of dosage unit, and adhesive strength or peel force); and metered dosage forms (number of discharges per container and delivered dose uniformity) [16].

#### 2.1.9. China

The Chinese government requires accelerated and long-term stability testing to provide shelf lives of herbal products and appropriate storage conditions [17]. The Anhui Provincial Food and Drug Administration (China) provides stability testing parameters for different forms of herbal products prescribed in the Chinese pharmacopeia, as follows: pills (description, identification, disintegration, water content, assay, and microbial limits); powders (description, identification, appearance uniformity, water content, particle size, assay, and for sterile powders used to topically treat wounds or burns or for external use and microbial limits); granules (description including moisture absorption and softening, identification, water content, dissolution, particle size, assay, and microbial limits); tablets (description, identification, hardness, disintegration, foaming capacity, assay, and microbial limits); concentrated decoctions (description including sucrose crystallization and phase separation, identification, relative density, insoluble material content, assay, and microbial limits); colloids (description, identification, water content, assay, and microbial limits); syrups (description, identification, relative density, pH, assay, and microbial limits); transdermals (description, identification, extractive in plaster mass, heat resistance, excipient property, adhesive property, and microbial limits); liquid mixtures (description including clarity, identification, relative density, pH, assay, and microbial limits); dripping pills (description, identification, disintegration, assay, and microbial limits); soft capsules (description, identification, disintegration, water content, assay, and microbial limits); medicinal wines (description, identification, ethanol content, methanol content, total solids, assay, and microbial limits); tinctures (description, identification, ethanol content, assay, and microbial limits); fluid extracts (description, identification, ethanol content, assay, and microbial limits); extracts (description, identification, assay, and microbial limits); plasters (description, identification, softening point, and assay); gels (description, identification, pH, viscosity, assay, and microbial limits); ointments (description including rancidification, odor, color, phase separation, identification, particle size, sterility for burn or wound treatments, and microbial limits); aromatic solutions (description, identification, pH, assay, and microbial limits); tea bags (description, identification, water content, solubilization, assay, and microbial limits); liniments, lotions, and smeared films (description, identification, relative density, pH, ethanol content, refractive index, and microbial limits); suppositories (description, identification, disintegration, assay, and microbial limits); nasal preparations (description, identification, pH, assay, sterility, and microbial limits); ophthalmic preparations (description, identification, pH, visible foreign matters, particle size, foreign metal contents, sterility, and microbial limits); aerosols (description, identification, delivery rate, total spray volume, total number of deliveries per container, quantity emitted per delivery, active ingredient content per delivery, particle size, sterility, and microbial limits); and sprays (description including precipitate and phase separation tendencies, identification, particle size, spray testing, assay, sterility, and microbial limits) [18].

#### 2.1.10. Egypt

Egyptian Drug Authority requires that *finished products* satisfy minimum specifications for registration, and these include the following: (1) common quality parameters: physical appearance (color, odor, form, shape, size, and texture), water content, identity tests, or qualitative determination of relevant substances of the plants (e.g., fingerprint chromatograms), quantification of relevant active ingredients, tests for residual solvents, other toxins, and microbiological contamination. (2) Specific quality parameters for dosage forms; tablets (uniformity of weight, disintegration time, hardness/friability for uncoated tablets, and dissolution); single-dose powders (uniformity of weight); suppositories (uniformity of weight and disintegration time); herbal tea in sachets (uniformity of weight); capsules (uniformity of weight, disintegration time, and dissolution); pills (disintegration time); internal and external fluids (viscosity); and semisolid preparations (consistency) [19].

#### 2.1.11. Hong Kong

The legislative council of Hong Kong prescribes that stability assessment of *proprietary Chinese medicines* is necessary to determine the shelf lives in sales packaging at room temperature or under proposed storage conditions, as described by “Product quality documents Technical guidelines” [20, 21].

The Hong Kong government provides stability testing parameters for various common dosage forms of *proprietary Chinese medicines*, as follows: injected mediciations (clarity, pH value, sterility, pyrogen, hemolysis, and irritation test); mixtures (clarity, relative density, and pH value); syrups (relative density and pH value); medicinal wines (ethanol content and total solids); pills (disintegration test and water content); powders (uniformity, water content, and degree of powder fitness); concentrated decoctions (description including tendency to crystallize and form layers, relative density, dissolution, and pH value); capsules and dripping pills (water content and disintegration); tablets (hardness and disintegration); liquid extracts (pH value, ethanol content, and total solids); granules (water content and size of granules); ointments (skin irritation); plasters (softening point and skin irritation); adhesive plasters (tension, skin irritation, and cold- and heat-proof); glues (water content); suppositories and troches (disintegration and pH value); aerosols (spraying efficacy, odor, and irritation); medicinal membrane (dissolution, irritation, and pH value); extracts; and suspensions. Description, identification (except for medicinal membrane), assay, and microbial limits (injected medications, plasters, and adhesive plaster are excluded) are generally required in all dosage forms [20].

#### 2.1.12. India

Indian government specifies the requirements for the quality test of herbal products used in Ayurvedic, Siddha, and Unani system of medicines according to dosage forms, as follows: tablets (description, identification, uniformity of weight, uniformity of diameter^*∗*^, disintegration test, and assay); capsules (description, identification, uniformity of weight, uniformity of diameter^*∗*^, disintegration test, and assay); and parental preparations (clarity, pH^*∗*^, identification, volume in container, sterility, pyrogen test^*∗*^, toxicity test^*∗*^, and assay). Asterisks indicate optional test parameters [22].

#### 2.1.13. Japan

The Pharmaceutical Safety and Environmental Health Bureau (Ministry of Health, Labor and Welfare) provides quality parameters for *Kampo* dosage forms, as follows: powders (content, description, identification, loss on drying, uniformity, and assay); granules (content, description, identification, loss on drying, uniformity, disintegration, and assay); uncoated and film-coated tablets (content, description, identification, loss on drying, uniformity, disintegration, and assay); sugar-coated tablets (content, description, identification, loss on drying, uniformity, and assay); and hard and soft capsules (content, description, identification, loss on drying, uniformity, disintegration, and assay) [23]. It should be noted that stability testing procedures should be conducted in accordance with ICH guidelines [24].

#### 2.1.14. Kenya

Kenyan government demands a minimum range of specifications be met by *finished products* according to guidelines for the registration of herbal and complementary products. General specifications include tests for microbiological contamination and toxins, physical appearance (color, odor, form, shape, size, and texture), water content, identity tests, qualitative determination, quantification of relevant active ingredients, and tests for residual solvents. Specific specifications for different dosage forms are as follows: tablets (uniformity of weight, disintegration time, hardness/friability for uncoated tablets, and dissolution), single-dose powders (uniformity of weight), suppositories (uniformity of weight and disintegration time), herbal tea in sachets (uniformity of weight), capsules (uniformity of weight, disintegration time, and dissolution), pills (disintegration time), internal and external fluids (viscosity), and semisolid preparations (consistency). The guideline prescribes that the physical and chemical stabilities after long-term storage period should comply with ICH guidelines [25].

#### 2.1.15. Republic of Korea

The Ministry of Food and Drug Safety (MFDS) requires the results of stability tests conducted in accordance with existing regulations, which provide long-term, accelerated, and intermediate testing for prescribed times under specified storage conditions, for the registration of herbal products [26, 27].

MFDS provides general specifications for all herbal dosage forms, which include description, identification, assay, and purity testing, and specific specifications for each dosage form: patches (disintegration, dissolution, alcohol content^*∗*^, adhesive strength, uniformity of dosage units, and texture^*∗*^); granules (microbial limits^*∗*^, disintegration, dissolution, particle-size distribution, and uniformity of dosage units); powders (microbial limits^*∗*^, disintegration^*∗*^, dissolution^*∗*^, particle-size distribution, and uniformity of dosage units); optic ointments (foreign metal particles, sterility, disintegration^*∗*^, dissolution^*∗*^, particle-size distribution, and uniformity of dosage units); liquids for internal use including lemonades, aromatic water, syrups, solutions, extracts, elixers, fluid extracts, emulsions, suspensions, decoctions, infusions, spirits, and tinctures (microbial limits, disintegration^*∗*^, dissolution^*∗*^, alcohol content^*∗*^, particle-size distribution^*∗*^, and uniformity of dosage units); liquids for external use including lotions, liniments, aromatic water, solutions, emulsions, and suspensions (microbial limits^*∗*^, disintegration^*∗*^, dissolution^*∗*^, alcohol content^*∗*^, particle-size distribution^*∗*^, and uniformity of dosage units); aerosols (microbial limits^*∗*^, alcohol content^*∗*^, particle-size distribution^*∗*^, and uniformity of dosage units); semisolids for external use including ointments, creams, and pastes (microbial limits, particle-size distribution^*∗*^, and uniformity of dosage units); ophthalmic solutions (sterility, insoluble particulates, insoluble foreign matters, disintegration^*∗*^, dissolution^*∗*^, particle-size distribution^*∗*^, and uniformity of dosage units); sprays (total amount of spray per delivery container, microbial limits, disintegration^*∗*^, dissolution^*∗*^, alcohol content^*∗*^, particle-size distribution^*∗*^, and uniformity of dosage units^*∗*^); tablets and capsules (microbial limits^*∗*^, disintegration^*∗*^, dissolution^*∗*^, and uniformity of dosage units); suppositories (microbial limits, disintegration, dissolution, and uniformity of dosage units); injections (sterility, insoluble particulates, insoluble foreign matters, disintegration^*∗*^, dissolution^*∗*^, endotoxin^*∗*^, pyrogens^*∗*^, particle-size distribution^*∗*^, and uniformity of dosage units); plasters and cataplasma (disintegration^*∗*^, dissolution^*∗*^, alcohol content^*∗*^, adhesive strength, and texture); troches (microbial limits^*∗*^, disintegration^*∗*^, dissolution^*∗*^, and uniformity of dosage units); and pills (microbial limits^*∗*^, disintegration^*∗*^, dissolution^*∗*^, and uniformity of dosage units^*∗*^) [26]. Asterisks indicate optional test parameters.

#### 2.1.16. The Philippines

The Food and Drug Bureau of the Philippines requires that stability studies be conducted under recommended conditions and should determine the most appropriate conditions for storage and shelf life. The government also requires physical descriptions, tests, and quality standards of *finished products* (herbal medicines and traditionally used herbal products) including organoleptic and macroscopic descriptions (appearance, texture, color, odor, and taste), moisture content, pH, alcohol content (if applicable), microbial limits, and identification. In addition, the Philippines government provides specific parameters of specifications for different dosage forms such as tablets (weight variation, content uniformity, disintegration, hardness test, friability, and microbial testing); capsules (weight variation, content uniformity, and microbial testing); syrups and liquids (viscosity, pH, and microbial testing); suspensions (suspendability, homogeneity, viscosity, minimum fill, pH, and microbial testing); ointments, creams, and semisolid preparations (palpability, homogeneity, pH, melting point, allergenicity, and microbial testing); suppositories and pessaries (allergenicity and microbial testing); and decoctions, infusions, extracts (liquids, pillulars, and powders), tinctures, syrups, lotions, and emulsions (must pass all requirements specified in Pharmacopoeias of other countries) [28, 29].

#### 2.1.17. Qatar

The Qatar government provides general requirements for the quality specifications of *herbal and dietary supplement products* for registration. These include physical examination, identification (chemical, spectroscopic, or chromatographic tests), main ingredient levels, heavy metals concentrations, microbial limits, and other quality standards according to dosage form, which include disintegration, dissolution, friability, hardness, water, pH, water content, ash, and residue on ignition tests. Applicable dosage forms are tablets, hard, and soft gelatin capsules, semisolid preparations (ointments, creams, and gels), herbal tea bags and sachets, syrups, oral suspensions, oral drops, or powders. General requirements of quality parameters for sterile products (eye drops, contact lenses, and dermal fillers) additionally include pH, osmolarity, viscosity, volume, and bacterial endotoxin test (for dermal filler products) [30].

#### 2.1.18. Switzerland

The Switzerland agency recommends that *bulk and finished medicinal products* in the form of capsules or tablets containing herbal preparations or granules require drug formulation-specific testing parameters (e.g., disintegration time and average weight). Stability tests are required to be conducted in accordance with international ICH guidelines, which include testing parameters such as description, identity, loss on drying, assay, and microbial purity [31].

#### 2.1.19. The United States of America

The Food and Drug Administration (FDA) requires that the stability of botanical drug substance and drug products be monitored using stability-related analytical methods or biological assay [32] and presents the quality testing attributes required for the registration of *botanical drug products* to ensure that clinical protocols are properly designed during phases 1, 2, and 3 clinical studies. General attributes include appearance, chemical identification, assay for active constituents or characteristic markers, biological assay (optional), strength by dry weight (of drug substance), and microbial limits and specific attributes of dosage forms (dissolution for solid oral products, sterility, nonpyrogenicity, and animal safety testing for parenterals) [33].

#### 2.1.20. Zambia

The Zambian government requires specifications and test methods of *final products* in all dosage forms comply with their guideline for the registration of herbal medicines, which includes description, identity, assay, and impurities (degradation product of active raw materials and microbial limits). Additional tests for specific dosage forms are also provided by the guideline, as follows: gelatin capsules and coated and uncoated tablets (dissolution, disintegration, hardness, friability, uniformity of dosage units, and water content); oral liquids (uniformity of contents, pH, microbial limits, antimicrobial and antioxidant preservative content, extractable from the container or closure system, alcohol content, dissolution for suspensions and powders for suspension, redispensability for suspensions, viscosity for suspensions or viscous solutions, specific gravity for suspensions or viscous solutions, and water content for powders for reconstitution) [34].

Considering global regulations and guidelines, testing parameters of oral or external dosage forms, which are commonly specified in more than two global guidelines, are classified into three groups: (1) physical parameters, e.g., description, purity, transparency, hardness, friability, water content, uniformity of dose units, weight variation, particle-size variation, viscosity, relative density, and resuspendability; (2) chemical parameters, e.g., assay, identification (by chromatographic fingerprinting in most cases), dissolution, disintegration, pH, and ethanol content; and (3) biological parameters, e.g., microbial limits, sterility, and irritation testing (Tables 1 and 2).

### 2.2. Stability Testing Methods

Global regulations for the stability testing of finished herbal products under long-term, accelerated, and intermediated conditions require that the frequency of stability studies be sufficient to establish a product's stability profile throughout its proposed shelf-life, especially for long-term stability testing. Herbal products should be evaluated in terms of thermal stability or moisture susceptibility based on consideration of durations of storage, transportation, and use. Moreover, the effects of storage temperature and moisture (relative humidity) should be adequately considered as they are the most influential factors for quality of herbal products. Herbal products are packed in either general, semipermeable (allows solvent or moisture migration through the container surface), or impermeable containers, which influence the effects of storage temperature and relative humidity [3, 7, 35, 36].

Long-term stability testing (=real-time stability testing), accelerated testing, and intermediate testing (if necessary) are usually undertaken according to established period to confirm the shelf-life of herbal products during the proposed testing period under storage conditions.

Long-term testing is carried out for less than 12 months by most authorities, though some including ASEAN, China, Hong Kong, Korea, and Zambia conduct long-term testing for more than 12 months. The storage test temperatures used are 25°C ± 2°C or 30°C ± 2°C with relative humidity of 60% ± 5%, 65% ± 5%, or 75% ± 5% (in general containers), or 35% ± 5% or 40% ± 5% (in semipermeable containers) under ambient storage conditions. For refrigerated products, the testing temperature used for general containers by most authorities is 5°C ± 3°C, except by Chinese authorities who adopt 6°C ± 2°C for general containers. All authorities use a freezing temperature of −20°C ± 5°C (Table 3).

Most global authorities and countries conduct accelerated testing for ≤6 months, except Korea (>6 months). Ambient testing temperature is 40°C ± 2°C with 75% ± 5% RH for in general container or <25% for in semipermeable containers in most cases. The Chinese guideline adds 30°C ± 2°C and 65% ± 5% RH for testing plasters, colloids, ointments, gels, ophthalmic ointments, suppositories, and aerosols. As regards refrigeration, temperatures of 25°C ± 2°C or 30°C ± 2°C and RHs of 60% ± 5%, 65% ± 5%, or 75% ± 5% are used for general containers. Only the Brazilian guideline states a freezing temperature of −20°C ± 5°C (Table 4).

Intermediate testing is conducted for ≤6 months (except for Korea >12 months) at 30°C ± 2°C by all authorities and an RH of 65% ± 5% for general and semipermeable containers or RH of 35% ± 5% for semipermeable containers as required by the EEC, WHO, and Republic of Korea (Table 5).

### 2.3. Stability Testing Parameters and Research Studies

Enayatifard et al. evaluated the microbial contaminations in solid dosage forms (tablets, powders, and capsules) with different packaging types and reported all samples were contaminated with *Salmonella* sp. and did not meet the microbial limit standard [38]. Guimarães et al. monitored the antioxidant activities of the decoctions and infusions of four medicinal herbs over different storage periods (0, 30, 60, and 120 days) and confirmed storage duration can influence the antioxidant activities and contents of different dosage forms [39]. Kim et al. tested the stability of a cream containing *Glycyrrhiza uralensis* extract by investigating pH, UV absorbance, viscosity, and color changes at different temperatures (4°C, 25°C, 37°C, and 45°C) and in sunlight for 12 weeks [40]. Pushpalatha et al. performed stability testing on steam-pasteurized Ashoka tablets by evaluating description, hardness, friability, weight variation, disintegration, polyphenol and catechin contents, TLC fingerprinting, and microbial limits (aerobic microbes, yeast, and mold) using real-time and accelerated testing conditions (0, 1, 2, 3, and 6 months) [41]. Sawant et al. formulated a Neem (*Azadirachta indica*) and Turmeric (*Curcuma longa*) extract containing ointment and evaluated color, odor, pH, spreadability, extrudability, consistency, diffusion, solubility, washability, and irritancy after storage at different temperatures (2°C, 25°C, and 37°C) over four weeks [42]. Alexander et al. measured the influence of a steaming treatment on the shelf-life of a sachets of xanthone-rich green herbal tea (*Cyclopia maculata* Andrews Kies) by testing sensory characteristics, color, and phenolic quality after storage at 0°C and 25°C for 6 months [43]. Huang et al. tested the physical and chemical stabilities of *Triphala* solution by measuring sediment formation and chromatographic profiles over 5 consecutive days [44], and Lee et al. tested the stability of a Mahwang-tang decoction by evaluating pH, total soluble solids, marker compound levels, and *in vitro* anti-inflammatory and antioxidant activities after storage at 4°C or room temperature for 3 months [45].

## 3. Concluding Remarks

Efforts by global authorities and countries to improve the qualities of herbal products continue to increase. However, the different regulations adopted inhibit the scopes of studies and harmonization of quality assessments of herbal products. In the present study, we provide stability testing parameters and methods and an overview of the guidelines and regulations of 5 global authorities and 15 countries. We hope that the information provided further understanding and collaborative studies on the stability testing of herbal products in their various dosage forms.

## Figures and Tables

**Table 1:** Common testing parameters of oral solid and liquid dosage forms of herbal products.

Parameter		Solid dosage form								Liquid dosage form								
		Capsules^a^	Extracts	Granules^b^	Herbal infusion bags^c^	Pills^d^	Powders^e^	Tablets^f^	Troches	Aromatic water	Decoction	Emulsion	Infusion	Fluid extracts^g^	Solution	Suspension	Syrup	Tincture
Physical	Description^1^	⋁	⋁	⋁	⋁	⋁	⋁	⋁	⋁	⋁	⋁	⋁	⋁	⋁	⋁	⋁	⋁	⋁
	Purity													⋁		⋁		
	Transparency/clarity														⋁			
	Hardness	⋁						⋁										
	Friability	⋁				⋁		⋁										
	Water content^2^	⋁	⋁	⋁	⋁	⋁	⋁	⋁						⋁		⋁		
	Uniformity of dose unit^3^	⋁		⋁	⋁	⋁	⋁	⋁						⋁		⋁	⋁	⋁
	Weight variation^4^	⋁						⋁										
	Particle-size variation^5^			⋁			⋁					⋁		⋁		⋁		
	Viscosity											⋁		⋁	⋁	⋁	⋁	
	Relative density																⋁	
	Resuspendability^6^															⋁		
	Reconstitution time															⋁		
	Dispersion^7^															⋁		
	Rheological properties															⋁		
	Phase separation											⋁						
	Polymorph conversion															⋁		

Chemical	Assay/content	⋁	⋁	⋁	⋁	⋁	⋁	⋁	⋁	⋁		⋁		⋁	⋁	⋁	⋁	⋁
	Identification	⋁	⋁	⋁	⋁	⋁	⋁	⋁	⋁	⋁	⋁	⋁	⋁	⋁		⋁	⋁	⋁
	Dissolution	⋁		⋁		⋁		⋁						⋁		⋁		
	Disintegration	⋁		⋁		⋁		⋁	⋁					⋁				
	pH	⋁	⋁									⋁		⋁	⋁	⋁	⋁	⋁
	Preservative content^8^															⋁		
	Ethanol content													⋁		⋁		⋁
	Extractable substances											⋁			⋁	⋁		

Biological	Microbial limit^9^	⋁	⋁	⋁	⋁	⋁	⋁	⋁	⋁	⋁	⋁	⋁	⋁	⋁	⋁	⋁	⋁	⋁

^1^Includes organoleptic characteristics such as color, odor, form, shape, size, and texture or absorption of moisture and the softening. ^2^Includes loss on drying. ^3^Includes uniformity of mass or uniformity of weight ^4^or average weight. ^5^Includes granule size variation, granule size distribution, or particle-size distribution ^6^or redispersibility ^7^or dispersibility. ^8^Includes antimicrobial and antioxidant preservative contents. ^9^Includes microbial content or microbial purity. ^a^Includes soft and hard gelatin capsules. ^b^Includes for oral solution or suspension. ^c^Includes herbal tea in sachets or herbal tea bags. ^d^Includes coated or uncoated pills, dripping pills, or pellets. ^e^Includes powders used for oral solutions or suspensions. ^f^Includes coated or uncoated tablets or sugar-coated tablets (pastilles). ^g^Includes liquid extract, internal fluid, oral liquid, or liquid mixture.

**Table 2:** Common testing parameters of external dosage forms of herbal products.

Parameter		Creams^a^	Emulsions	Gels^a^	Liniments	Lotions^a^	Ointments^a^	Solutions^a^	Suspensions^a^	External fluid	Suppositories^b^	Injections	Plasters^c^	Transdermal patches	Inhalers	Sprays^d^	Aerosols
Physical	Description^1^	⋁	⋁		⋁	⋁	⋁	⋁			⋁	⋁	⋁	⋁		⋁	⋁
	Purity							⋁					⋁				
	Transparency/clarity	⋁				⋁	⋁					⋁					
	Homogeneity	⋁				⋁	⋁										
	Particulate matter							⋁									
	Microscopic evaluation														⋁	⋁	
	Foreign mechanical inclusions														⋁		
	Weight variation^2^	⋁				⋁	⋁	⋁			⋁				⋁	⋁	
	Uniformity of dose unit^3^	⋁						⋁			⋁			⋁	⋁		
	Particle-size distribution	⋁				⋁	⋁	⋁	⋁								
	Aerodynamic particle-size distribution														⋁	⋁	
	Water content^4^										⋁				⋁	⋁	
	Softening temperature										⋁		⋁				
	Viscosity	⋁		⋁		⋁	⋁			⋁							
	Adhesive forces^5^												⋁	⋁			
	Pump delivery														⋁	⋁	

Chemical	Assay/content						⋁				⋁		⋁			⋁	⋁
	Identification		⋁		⋁	⋁	⋁	⋁			⋁	⋁	⋁	⋁		⋁	⋁
	Dissolution							⋁			⋁		⋁				
	Disintegration							⋁			⋁		⋁				
	pH	⋁		⋁		⋁	⋁				⋁	⋁					
	Extractable volume	⋁					⋁	⋁	⋁								
	Ethanol content				⋁	⋁							⋁				
	Extractables/leachables from devices^6^														⋁		

Biological	Allergenicity/primary irritation test										⋁		⋁				
	Sterility	⋁					⋁	⋁	⋁			⋁		⋁			
	Pyrogenicity^7^											⋁					
	Microbiological limits^8^	⋁	⋁	⋁	⋁	⋁	⋁	⋁	⋁		⋁		⋁	⋁	⋁	⋁	⋁
	Release rate *in vitro*													⋁			

^1^Includes organoleptic characteristics such as color, odor, form, shape, size, and texture; rancidification, odor, color, phase separation, and coating for ointments; and precipitate and phase separation for sprays. ^2^Includes weight loss or average weight. ^3^Includes uniformity of mass or uniformity of weight ^4^or moisture content. ^5^Includes adhesive strength, adhesive property, peel force, peel strength, or tensile strength. ^6^Includes substances extracted and discharged from plastic and elastomeric components of the container, closure, and pump. ^7^Includes the content of endotoxins. ^8^Includes microbial content or microbial purity. ^a^Includes dosage form for ophthalmic or otic use. ^b^Includes intravaginal suppositories (or pessaries). ^c^Includes cataplasma or medicinal membrane. ^d^Includes dosage form for nasal use.

**Table 3:** Storage conditions used for long-term stability testing.

Global community	Testing period (container type)	Storage conditions (temperature/relative humidity, RH)		
		In ambient storage	In refrigerator	In freezer
ASEAN [5]	0, 3, 6, 9, 12, 18, 24 months, and annually thereafter	30°C ± 2°C/75% RH ± 5% RH (moisture-permeable container^1^) 30°C ± 2°C (moisture-impermeable container^2^)	5°C ± 3°C	—

EEC [7]	12 months^a^ (general container) 6 or 12 months^b^ (general container)	25°C ± 2°C/60% RH ± 5% RH or 30°C ± 2°C/65% RH ± 5% RH or 30°C ± 2°C/75% RH ± 5% RH	5°C ± 3°C	-20°C ± 5°C
	12 months (semipermeable container^3^)^a^ 6 or 12 months (semipermeable container)^b^	25°C ± 2°C/40% RH ± 5% RH or 30°C ± 2°C/35% RH ± 5% RH	—	—

EMA [35]	6 or 12 months (general container)	30°C ± 2°C/60% RH ± 5% RH (6 months) 30°C ± 2°C/65% RH ± 5% RH (12 months)	5°C ± 3°C	-20°C ± 5°C
	6 or 12 months (semipermeable container)	25°C ± 2°C/40% RH ± 5% RH (6 months) 30°C ± 2°C/35% RH ± 5% RH (12 months)	—	—

ICH [36]	12 months (general container)	25°C ± 2°C/60% RH ± 5% RH or 30°C ± 2°C/65% RH ± 5% RH	5°C ± 3°C	−20°C ± 5°C
	12 months (semipermeable container)	25°C ± 2°C/40% RH ± 5% RH or 30°C ± 2°C/35% RH ± 5% RH	—	—

WHO [3]	6 or 12 months (general case)	25°C ± 2°C/60% RH ± 5% RH or 30°C ± 2°C/65% RH ± 5% RH or 30°C ± 2°C/75% RH ± 5% RH	5°C ± 3°C	−20°C ± 5°C
	6 or 12 months (semipermeable case)	25°C ± 2°C/40% RH ± 5% RH or 30°C ± 2°C/35% RH ± 5% RH	—	—

Australia [12]	12 months (general container)	25°C ± 2°C/60% RH ± 5% RH or 30°C ± 2°C/65% RH ± 5% RH	5°C ± 3°C	−20°C ± 5°C
	12 months (semipermeable container)	25°C ± 2°C/40% RH ± 5% RH or 30°C ± 2°C/35% RH ± 5% RH	—	—

Brazil [37]	12 months (impermeable container)	30°C ± 2°C	5°C ± 3°C	−20°C ± 5°C
	12 months (semipermeable container)	30°C ± 2°C/75% RH ± 5% RH	5°C ± 3°C	−20°C ± 5°C

China [17, 18]	0, 3, 6, 9, 12, and 18 months (24 and 36 months, if necessary)	25°C ± 2°C/60% RH ± 10% RH or 30°C ± 2°C/65% RH ± 10% RH	6°C ± 2°C	—

Hong Kong [21]	Every month for 3 consecutive months initially and then every 6 months	25°C ± 2°C/60% RH ± 5% RH	—	—

Japan [24]	12 months (general container)	25°C ± 2°C/60% RH ± 5% RH or 30°C ± 2°C/65% RH ± 5% RH	5°C ± 3°C	−20°C ± 5°C
	12 months (semipermeable container)	25°C ± 2°C/40% RH ± 5% RH or 30°C ± 2°C/35% RH ± 5% RH	—	—

Kenya [25]	12 months (general container)	25°C ± 2°C/60% RH ± 5% RH or 30°C ± 2°C/65% RH ± 5% RH	5°C ± 3°C	−20°C ± 5°C
	12 months (semipermeable container)	25°C ± 2°C/40% RH ± 5% RH or 30°C ± 2°C/35% RH ± 5% RH	—	—

Korea [27]	0, 3, 6, 9, 12, 18, 24 months, and annually thereafter (general container)	25°C ± 2°C/60% RH ± 5% RH or 30°C ± 2°C/65% RH ± 5% RH	5°C ± 3°C	−20°C ± 5°C
	0, 3, 6, 9, 12, 18, 24 months, and annually thereafter (semipermeable container^4^)	25°C ± 2°C/40% RH ± 5% RH or 30°C ± 2°C/35% RH ± 5% RH	—	—

Philippines [28, 29]	—	30°C ± 2°C/75% RH ± 5% RH	—	—

Zambia [34]	3, 6, 9, 12, 18, 24, and 36 months	25°C ± 2°C/65% RH ± 5% RH	—	—

^a^New medicinal products. ^b^Medicinal products manufactured from the existing pharmaceutical substances. ^1^Includes polyvinyl chloride (PVC) blisters, low-density polyethylene (LDPE) bottles, and glass or HDPE bottles when fitted with polypropylene closures. ^2^Includes aluminum/aluminum blisters, high-density polyethylene (HDPE) or glass bottles fitted with metal or HDPE closures. ^3^Includes plastic bags and soft low-density polyethylene bags for parenteral medicinal products of large volumes, as well as ampoules and vials made of low-density polyethylene. ^4^Includes plastic bags, semisolid low-density polyethylene bags or low-density polyethylene samples, bottles, or vials.

**Table 4:** Storage conditions used for accelerated stability testing.

Global community	Testing period (container type)	Storage condition (temperature/relative humidity, RH)		
		In ambient storage	In refrigerator	In freezer
ASEAN [1]	0, 3, and 6 months (including the initial and final time points)	40°C ± 2°C/75% RH ± 5% RH	25°C ± 2°C/60% RH ± 5% RH	—

EEC [7]	6 months (general container)	40°C ± 2°C/75% RH ± 5% RH	25°C ± 2°C/60% RH ± 5% RH or 30°C ± 2°C/65% RH ± 5% RH or 30°C ± 2°C/75% RH ± 5% RH	—
	6 months (semipermeable container)	40°C ± 2°C/not more than 25% RH	—	—

EMA [35]	6 months (general container)	40°C ± 2°C/75% RH ± 5% RH	25°C ± 2°C/60% RH ± 5% RH	—
	6 months (semipermeable container)	40°C ± 2°C/not more than 25% RH	—	—

ICH [36]	6 months (general container)	40°C ± 2°C/75% RH ± 5% RH	25°C ± 2°C/60% RH ± 5% RH	—
	6 months (semipermeable container)	40°C ± 2°C/not more than 25% RH	—	

WHO [3]	6 months (general case)	40°C ± 2°C/75% RH ± 5% RH	25°C ± 2°C/60% RH ± 5% RH or 30°C ± 2°C/65% RH ± 5% RH or 30°C ± 2°C/75% RH ± 5% RH	—
	6 months (semipermeable case)	40°C ± 2°C/not more than 25% RH	—	—

Australia [12]	6 months (general container)	40°C ± 2°C/75% RH ± 5% RH	25°C ± 2°C/60% RH ± 5% RH	—
	6 months (semipermeable container)	40°C ± 2°C/not more than 25% RH	—	

Brazil [37]	6 months (impermeable container)	40°C ± 2°C	25°C ± 2°C	−20°C ± 5°C
	6 months (semipermeable container)	40°C ± 2°C/75% RH ± 5% RH	25°C ± 2°C/60% RH ± 5% RH	−20°C ± 5°C

China [17, 18]	0, 1, 2, 3, and 6 months (general container)	40°C ± 2°C/75% RH ± 5% RH	25°C ± 2°C/60% RH ± 5% RH	—
	0, 1, 2, 3, and 6 months (semipermeable container^a^)	40°C ± 2°C/20% RH ± 5% RH	—	—
	0, 1, 2, 3, and 6 months^b^	30°C ± 2°C/65% RH ± 5% RH	—	—

Hong Kong [21]	Every month for 3 consecutive months	37°C–40°C/75% RH ± 5% RH	—	—

Japan [24]	6 months (general container)	40°C ± 2°C/75% RH ± 5% RH	25°C ± 2°C/60% RH ± 5% RH	—
	6 months (semipermeable container)	40°C ± 2°C/not more than 25% RH	—	—

Korea [27]	More than 6 months (general container)	40°C ± 2°C/75% RH ± 5% RH	25°C ± 2°C/60% RH ± 5% RH	—
	More than 6 months (semipermeable container)	40°C ± 2°C/not more than 25% RH	—	—

Philippines [28, 29]	—	40°C ± 2°C/75% RH ± 5% RH	—	—

Zambia [34]	0, 1, 2, 3, and 6 months	40°C ± 2°C/75% RH	—	—

^a^Includes multilayer coextrusion polyvinyl chloride soft bag injection and plastic bottle containing ophthalmic solution or nasal solution. ^b^Includes plasters, colloids, ointments, gels, ophthalmic ointments, suppositories, and aerosols.

**Table 5:** Storage conditions used for intermediate stability testing.

Global community	Testing period (container type)	Storage condition (temperature/relative humidity, RH)
EEC [7]	6 months (general container)	30°C ± 2°C/65% RH ± 5% RH
	6 months (semipermeable container)	30°C ± 2°C/35% RH ± 5% RH
EMA [35]	6 months (general container)	30°C ± 2°C/65% RH ± 5% RH
	6 months (semipermeable container)	30°C ± 2°C/65% RH ± 5% RH
ICH [36]	6 months (general container)	30°C ± 2°C/65% RH ± 5% RH
	6 months (semipermeable container)	30°C ± 2°C/65% RH ± 5% RH
WHO [3]	6 months (general case)	30°C ± 2°C/65% RH ± 5% RH
	6 months (semipermeable case)	30°C ± 2°C/35% RH ± 5% RH
Australia [12]	6 months (general container)	30°C ± 2°C/65% RH ± 5% RH
	6 months (semipermeable container)	30°C ± 2°C/65% RH ± 5% RH
China [17, 18]	0, 1, 2, 3, and 6 months	30°C ± 2°C/65% RH ± 5% RH
Japan [24]	6 months (general container)	30°C ± 2°C/65% RH ± 5% RH
	6 months (semipermeable container)	30°C ± 2°C/65% RH ± 5% RH
Korea [27]	More than 12 months (general container)	30°C ± 2°C/65% RH ± 5% RH
	More than 12 months (semipermeable container)	30°C ± 2°C/35% RH ± 5% RH
